# Acceptable risk as a psychological judgment: risk perception, trust, and fairness

**DOI:** 10.3389/fpsyg.2026.1868729

**Published:** 2026-06-22

**Authors:** Yiju Tang, Junjie Wei, Haoyang Ren

**Affiliations:** 1 College of Municipal and Environmental Engineering, Henan University of Urban Construction, Pingdingshan, China; 2 School of Intelligent Engineering, Shandong Management University, Jinan, China

**Keywords:** acceptable risk, fairness, psychological judgment, risk perception, trust

## Abstract

Acceptable risk is often defined in technical, regulatory, or ethical terms. Yet people do not decide that a risk is acceptable simply by estimating probabilities or expected harms. Judgments of acceptability are also shaped by how a hazard is perceived, whether those responsible for managing it are trusted, and whether exposure is regarded as fair. This mini review brings together research on risk perception, trust, and fairness to show how acceptable-risk judgments are formed within interpersonal configurations of exposure, control, and justification. The framework distinguishes primarily self-regarding risks, other-controlled risks, and collectively governed or mandated risks, arguing that these configurations affect the relative salience of perceived threat, institutional credibility, and legitimacy. This perspective helps explain why technically similar risks may be tolerated, contested, or rejected across settings. It also identifies implications for future research on risk communication, public judgment, and the psychology of legitimacy.

## 1 Introduction

Acceptable risk is usually discussed as a threshold to be defined, a regulatory category, or an ethical conclusion about how much danger may reasonably be tolerated. Such accounts are necessary, but they do not fully explain how acceptability is judged in everyday and institutional settings. Public responses rarely follow probability estimates alone (Fischhoff et al., 1978; Slovic, 1987; Siegrist and Árvai, 2020). People also ask whether a hazard feels threatening, whether those managing it can be relied upon, and whether exposure has been imposed on fair terms. In this review, risk acceptance refers to a judgment that exposure is sufficiently justified to be lived with, permitted, or supported. Risk tolerance is treated as a weaker and more conditional stance: people may endure or put up with a risk without positively endorsing it. Thus, tolerance can be one possible outcome of acceptable-risk judgment, but it does not exhaust acceptance. In this sense, acceptable risk should also be understood as a psychological judgment. Risks become acceptable, tolerable, contested, or rejected through interpretation as well as calculation (Loewenstein et al., 2001; Fox-Glassman and Weber, 2016).

Existing discussions of acceptable risk tend to follow two broad lines. One treats acceptable risk mainly as a matter of technical estimation, regulatory threshold-setting, or ethical justification. In this review, ethical reasoning is not treated as a component of risk perception; rather, it provides a normative language for asking whether exposure can be justified. A second line of research emphasizes public judgment, institutional trust, and perceived fairness in shaping whether exposure is accepted in practice (Balog-Way et al., 2020; Siegrist, 2021). Perceived fairness and legitimacy are therefore treated here as psychological and social forms through which normative concerns about justified exposure enter public acceptability judgments.

Building primarily on the latter line of research, this mini review brings together three related bodies of work. Research on risk perception shows that people evaluate hazards through qualities such as dread, familiarity, controllability, and catastrophic potential, not probability alone (Siegrist and Árvai, 2020). Work on trust suggests that risk judgments are shaped by confidence in those who regulate, communicate, and manage exposure (Siegrist, 2021; Pauer et al., 2024). Research on fairness and legitimacy, especially in the procedural-justice tradition, shows that acceptance depends not only on outcomes, but also on whether decision processes are seen as respectful, transparent, and normatively appropriate (Chan et al., 2025).

The review also adds a further issue: the interpersonal configuration of the risk situation. Acceptability is not judged in the same way when exposure is primarily self-regarding, when exposure is controlled by other actors, or when a risk is imposed, regulated, or justified through collective decision-making. These configurations differ in who is exposed, who decides, where risk control is located, and how the terms of exposure are justified. Specifying this configuration helps clarify how perceived threat, institutional credibility, and legitimacy become more or less salient across settings.

## 2 Risk perception and the felt meaning of hazard

Any psychological account of acceptable risk has to start with risk perception. Before people decide whether they can live with a risk, they form some sense of what sort of danger it is. That judgment is not reducible to a numerical estimate. Classic studies showed that responses to hazards vary with qualitative features such as dread, catastrophic potential, familiarity, voluntariness, controllability, and the visibility or delay of harms (Fischhoff et al., 1978; Slovic, 1987). Probability matters, but so does the kind of event people think the hazard would be.

This point matters directly for judgments of acceptability. A hazard may be low in statistical probability and still be experienced as unacceptable if it is involuntary, poorly understood, catastrophic, or beyond personal control (Balžekienė et al., 2025). By contrast, a relatively high level of exposure may be tolerated when it is familiar, routine, and woven into everyday practice. Acceptability is shaped, then, by the perceived character of a hazard as well as by its formal assessment. Evidence is filtered through cues that shape the perceived meaning of danger.

Acceptability judgments are therefore qualitative as well as probabilistic. Affect matters because people often rely on immediate positive or negative impressions when judging risks and benefits; hazards that evoke aversion tend to be seen as riskier and less beneficial (Efendić et al., 2022; Waters et al., 2023). Affect can register prior experience, symbolic meaning, and anticipated consequence, rather than merely distorting judgment (Karlsson et al., 2023). A condition that feels disturbing or ominous may therefore be judged more threatening even when technical assessments emphasize low probability. When the objection concerns whether exposure is morally justified or fairly imposed, however, the issue moves beyond risk perception and into fairness and legitimacy.

Uncertainty deepens this problem. Ambiguity, expert disagreement, and unstable guidance can intensify perceptions of risk because they widen the range of possible threat (Schneider et al., 2022; Johnson et al., 2024; Vromans et al., 2024). A hazard may be tolerated when its limits are known, but less so when people suspect that those managing it cannot define its boundaries or revise decisions in time. Risk perception therefore reflects the hazard itself, the social interpretation of risk signals, and the ways those signals are amplified (Perlstein, 2024).

Risk perception alone does not explain why some forms of exposure are tolerated while others are resisted (Priolo et al., 2025). Hazards that are regarded as serious may nonetheless remain tolerable when those responsible for managing them are seen as competent and responsive. Conversely, even moderate hazards may become unacceptable under conditions of institutional distrust. Perception is therefore an important part of acceptable-risk judgment, but not a sufficient account of its variation across settings.

## 3 Trust and the perceived governability of risk

Trust becomes especially important where knowledge is limited, consequences are uncertain, and dependence on institutions is unavoidable. In most contemporary risk settings, people do not assess hazards directly. They rely on experts, regulators, organizations, and communicators to define problems, monitor change, and intervene when conditions shift (Siegrist, 2021; Besley et al., 2026). Judgments of acceptable risk are therefore also judgments about whether those actors can be relied upon to manage exposure responsibly (Palomo-Vélez et al., 2023; Vrieling et al., 2023).

The relevance of trust depends on how exposure and control are distributed. In self-regarding risks, trust may operate mainly as confidence in one’s own judgment, skill, or information sources. In other-controlled risks, however, exposure and control are separated. Affected people must rely on employers, regulators, experts, organizations, or technological systems to keep exposure within acceptable bounds. Here, trust becomes a condition under which dependence on others can be experienced as tolerable.

Trust should not be treated as something added after hazard appraisal. In many cases, it shapes the judgment from the outset. Confidence in institutions and managing authorities is closely related to perceived risk, perceived benefit, and public acceptance of hazardous activities (Siegrist, 2021; Siegrist et al., 2021; Pauer et al., 2024). When people lack detailed technical knowledge, social trust becomes a cue for whether a hazard is manageable. What matters is whether those responsible for managing the hazard are regarded as competent, honest, and attentive enough to be believed (Hautea et al., 2024; Cologna et al., 2025).

Trust is also differentiated. Competence-based trust refers to the belief that those responsible for managing a risk have the expertise, resources, and practical capacity needed to identify, monitor, and mitigate the hazard. Integrity-based trust refers to the belief that these actors are honest, transparent, and guided by acceptable motives rather than by manipulation, concealment, or disregard for affected people (Liu et al., 2022; Hautea et al., 2024; Besley et al., 2026). These two forms of trust may diverge. People may regard an institution as technically capable while doubting its motives, or they may believe that actors are well intentioned while doubting their ability to control the hazard. Neither competence nor integrity is sufficient on its own. Public acceptance is unlikely to hold if those responsible appear incapable, if they are suspected of withholding information, or if their decisions seem unresponsive to those who bear the risk. Competence-based and integrity-based trust may therefore shape later judgments of acceptability rather than merely reflect them (Liu et al., 2022; Palomo-Vélez et al., 2024).

Even so, trust does not settle acceptability on its own. People may trust expertise and still object to how burdens are imposed or distributed. Trust may support acceptance, but questions of legitimacy remain, especially where exposure is seen as unfair.

## 4 Fairness and the legitimacy of exposure

Fairness directs attention to whether exposure is organized on terms people regard as legitimate. People do not judge risks solely by asking whether a danger is serious or whether authorities are capable. They also ask whether it is reasonable that they, rather than others, should bear the burden, under what conditions that burden has been imposed, and whether the decision process has treated them with respect. Judgments of acceptable risk are therefore cognitive and institutional, but also moral and relational (Bergquist, 2025; Moon and Travaglino, 2025).

Fairness becomes especially salient when risk is not confined to a single primarily self-regarding exposure. When exposure affects multiple people, when some actors benefit while others bear the burden, or when authorities decide on behalf of affected groups, acceptability becomes a question of legitimate social organization. People then ask not only whether the risk is serious or manageable, but whether they have been included, whether reasons have been given, and whether burdens are proportionate.

One aspect of fairness concerns distribution. People are sensitive to who bears risks and who receives benefits (Kollmann et al., 2024). Exposure is more likely to be resisted when burdens fall disproportionately on groups with less power or fewer resources while benefits accrue elsewhere. A hazard may be described as low in aggregate terms and still be experienced as unacceptable when the burden is concentrated, cumulative, or imposed on those least able to refuse it (Liu et al., 2021; Kollmann et al., 2024).

A second aspect concerns procedure. Research on procedural justice shows that people care not only about outcomes, but also about whether decision processes signal neutrality, transparency, respect, and voice (Dierckx et al., 2024). People may tolerate difficult or uncertain conditions when procedures are open, reasons are given, and concerns can be expressed without dismissal (Ndi, 2024; Shejale et al., 2025). Even when substantive outcomes remain unchanged, perceived unfairness in process can weaken acceptance because it undermines the sense that exposure is governed legitimately (Yokoyama et al., 2023; Takada et al., 2025).

Fairness also links risk judgment to emotion. Perceived injustice often produces anger, resentment, humiliation, and moral refusal rather than simple concern (Gregersen et al., 2023; Jansma et al., 2024). Once exposure is interpreted as unfair, resistance is no longer well explained as misunderstanding or informational deficit. Recent studies of waste-to-energy and waste-management acceptance similarly indicate that trust, reputation, fairness, risk perception, and acceptance are interwoven rather than separable considerations (Uji et al., 2025; Nuortimo et al., 2026).

## 5 Integrating perception, trust, fairness, and interpersonal configuration

**Figure 1:**
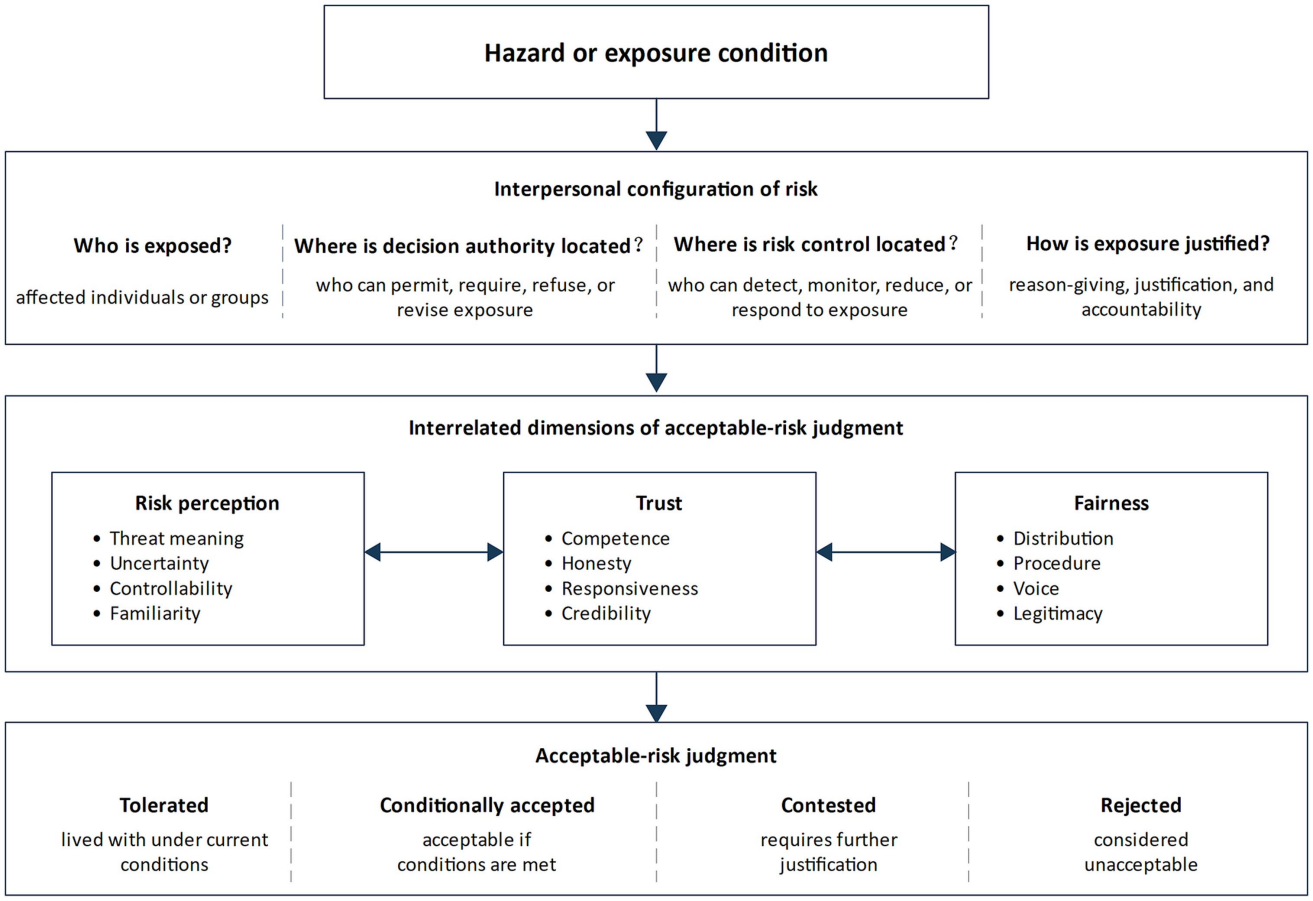
Acceptable risk as a psychological judgment: an integrative framework. Acceptable-risk judgments are shaped by hazard or exposure conditions, but these conditions are interpreted within interpersonal configurations of exposure, the location of decision authority, the location of risk control, and the justification of exposure. Risk perception, trust, and fairness interact to shape whether a risk is tolerated, conditionally accepted, contested, or rejected.

Risk perception, trust, and fairness can be distinguished analytically, but they should not be read as three independent predictors of acceptance. They interact in the formation of acceptable-risk judgments. Risk perception concerns what kind of danger an exposure condition is taken to represent. Trust concerns whether those who define, communicate, and manage that danger are regarded as capable, honest, and responsive. Fairness concerns whether the burdens, benefits, and decision procedures surrounding exposure are regarded as legitimate. Figure 1 brings these relationships together.

The interaction among the three dimensions becomes clearer when the interpersonal configuration of risk is specified. In primarily self-regarding risks, the main expected benefits and burdens fall on the same individual, who has substantial discretion over whether and how to engage in the activity. This category is not limited to deliberate risk-taking; it also includes ordinary activities with residual hazards, such as swimming in open water, driving, or personal health-related choices. The key point is that exposure, choice, and precaution are mainly located in the same individual. In such cases, perceived severity, familiarity, controllability, and self-efficacy may carry particular weight. Trust and fairness are not absent, but they often operate indirectly, for example through trust in information sources or confidence in one’s own capacity to manage the hazard, or the perceived fairness of background conditions that shape individual choice. In other-controlled risks, exposure and control are separated. Affected people must rely on experts, employers, regulators, organizations, or technical systems to identify, monitor, and mitigate the hazard. In these settings, trust becomes central because acceptability depends on whether dependence on others is experienced as warranted. In collectively governed or mandated risks, the question is not only whether a hazard is serious or manageable, but whether the distribution of burdens and the decision process can be justified to those affected. Here, fairness and legitimacy move to the foreground. In this sense, interpersonal configuration specifies both the scope of those affected and the location of decision authority, rather than functioning as an additional predictor alongside perception, trust, and fairness.

This configuration-based view helps explain why technically similar risks may be judged differently across settings. A hazard that is perceived as serious may still be tolerated when it is controlled by trusted institutions and governed through procedures regarded as fair. Conversely, a moderate exposure may become unacceptable when affected groups distrust the actors in control or view the distribution of burden as unjust. The physical magnitude of the hazard may be similar, but the social organization of exposure changes the judgment. Acceptability therefore depends not only on what the risk is, but also on who is asked to bear it, where risk control is located, and how the arrangement is justified.

The framework also clarifies several interaction mechanisms. Uncertainty can increase reliance on trust because people need credible actors to interpret incomplete or contested evidence. Trust can make uncertainty appear manageable rather than alarming, but distrust can make the same uncertainty appear as evidence of concealment, incompetence, or indifference (Schug et al., 2024). Fairness can stabilize acceptance when outcomes remain uncertain, because transparent procedures, voice, and review mechanisms can make provisional exposure more legitimate. By contrast, unfair treatment can turn even a technically moderate risk into a legitimacy conflict. In this sense, risk perception, trust, and fairness are not sequential stages. They mutually shape whether exposure is interpreted as threatening, governable, and justified.

For future empirical research, this means that acceptable risk should not be treated simply as the inverse of perceived risk or as the predictable outcome of better information. Future research should specify whether the exposed person is also the decision-maker, whether risk is controlled by other actors, whether burdens and benefits are distributed across groups, and whether affected people have procedural voice. This would help explain why the same level of perceived hazard may generate different degrees of acceptance across public health, workplace safety, environmental governance, energy transition, AI governance, and other domains.

In practice, the framework is useful as a diagnostic tool because rejection may arise from perceived threat, distrust, or unfairness. Threat-driven rejection calls for clearer explanation of uncertainty, consequence, and controllability. Distrust requires demonstrated competence, honesty, and responsiveness, not additional information alone. Unfairness requires attention to burden distribution, participation, review procedures, and contestability. Acceptance is not secured by reassurance alone; it depends on whether the risk is made intelligible, governable, and justifiable to those who bear it (Balog-Way et al., 2020; Siegrist, 2021; Ndi, 2024; Shejale et al., 2025).

## 6 Discussion

The central claim of this mini review is that acceptable risk cannot be settled by technical estimation or ethical justification alone, although both remain necessary. It is also a psychological judgment shaped by how danger is interpreted, whether those managing it are regarded as credible, and whether the terms of exposure are seen as fair. The revised framework adds that these dimensions operate within interpersonal configurations of risk. Acceptability is judged differently when exposure is primarily self-regarding, when it is controlled by other actors, or when it is collectively imposed and justified through policy, regulation, or institutional decision-making. A risk that appears threatening may still be tolerated when managing actors are trusted and procedures are regarded as legitimate. Conversely, a technically moderate risk may become contested or rejected when exposure is imposed without trust, procedural voice, or fair burden distribution (Dryhurst et al., 2020; Siegrist, 2021; Brauner et al., 2025; Görsch et al., 2025).

Future research should therefore avoid treating risk perception, trust, and fairness as context-free predictors of acceptance. Their relative importance is likely to depend on whether exposure, control, and decision authority are located in the same person, separated across actors, or organized through collective decision-making. One unresolved issue concerns the temporal ordering of trust, risk perception, fairness, and acceptability. Trust may precede risk perception when people lack technical knowledge, but it may also be shaped by prior controversies, institutional conduct, and the handling of uncertainty (Palomo-Vélez et al., 2023; Pauer et al., 2024). Similarly, procedural and distributive fairness should be examined separately because some settings turn mainly on participation and respectful treatment, whereas others turn on who receives benefits and who bears burdens (Liu et al., 2021; Zhang and Lin, 2023; Dierckx et al., 2024; Kollmann et al., 2024; Hampton-Smith et al., 2025).

Practically, the framework cautions against treating public concern as a simple information deficit. Institutions seeking acceptance need to identify whether rejection reflects perceived threat, distrust, or perceived unfairness. These problems require different responses: clearer explanation of uncertainty and consequence may help when threat perception dominates; visible competence, honesty, responsiveness, and accountability are needed when distrust is central; and participation, burden distribution, review procedures, and contestability matter when concerns are rooted in unfairness (Balog-Way et al., 2020; Siegrist, 2021; Schneider et al., 2022; Johnson et al., 2024; Ndi, 2024; Shejale et al., 2025). Framed in this way, acceptable risk becomes a useful meeting point for research on risk perception, institutional trust, fairness, and the psychology of legitimacy.
